# Recent and historical data show no evidence of Pacific bluefin tuna reproduction in the southern California Current system

**DOI:** 10.1371/journal.pone.0269069

**Published:** 2022-05-26

**Authors:** Heidi Dewar, Owyn E. Snodgrass, Barbara A. Muhling, Kurt M. Schaefer

**Affiliations:** 1 NOAA Fisheries, Southwest Fisheries Science Center, La Jolla, CA, United States of America; 2 Institute of Marine Sciences, University of California, Santa Cruz, CA, United States of America; 3 Inter-American Tropical Tuna Commission, La Jolla, CA, United States of America; Universita degli Studi di Bari Aldo Moro, ITALY

## Abstract

Despite their broad distribution across the North Pacific Ocean, the only known spawning grounds for Pacific Bluefin Tuna (*Thunnus orientalis*) are around coastal Japan and the East China Sea. However, an increase in the prevalence of large bluefin tuna up to 10 years old in the California Current System during exceptionally warm ocean conditions has led to speculation that they may be spawning in this region. To investigate this possibility, we collected samples from 36 females (estimated 3–8 years old) between 2015 and 2019. Histological analyses revealed no signs of imminent, active, or recent spawning. Further examination of historical ichthyoplankton collections showed no records of larval bluefin tuna, but confirmed the presence of the larvae of other tuna species in waters > 24°C. Fishery-dependent records showed that bluefin tuna are rarely recorded in purse seine catches where surface temperatures exceed 23°C. Our study, therefore, provided no evidence of bluefin tuna reproduction in the CCS. However, more comprehensive sampling, in particular off southern Baja California, may be required to confirm the absence of spawning.

## Introduction

Bluefin tunas (family Scombridae) are broadly distributed in tropical and temperate waters with three species occurring globally: Atlantic bluefin (*Thunnus thynnus*), Southern bluefin (*T*. *maccoyii*) and Pacific bluefin (PBF, *T*. *orientalis*). Unlike the tropical tunas, bluefin tunas spawn in restricted geographic areas within defined spawning seasons [1, 2]. PBF are known to spawn only in the Sea of Japan, the East China Sea, and off the Pacific coast of Japan [3, 4], with most larvae are collected where sea surface temperatures (SST) are 24–28°C [5]. PBF spawning in the Sea of Japan are younger (3–8 years old), while spawners in the East China Sea are typically 8 years of age or older [6, 7]. The current stock assessment [8] considers that 100% of PBF are mature at age five.

A portion of juvenile PBF migrate to the California Current System (CCS) to forage in their first or second year, and typically remain for 2–3 years before returning to the western Pacific [9]. Consequently, PBF landed by CCS fisheries have historically been relatively young (1–3 years old). However, in the late 1970s many 4–6 year old PBF were caught using purse seines [10], and fish approximately 4–8 years old were landed by recreational fishers in 1994 [11]. Since 2014, larger PBF have again been reported in CCS fisheries, with some potentially as old as 10 [12].

Spawning of PBF has never been reported in the CCS. However, large PBF caught in recent years are similar sizes to western Pacific spawners [6, 7], leading to speculation that PBF may be maturing and spawning in the CCS. Other lines of evidence support the plausibility of this hypothesis. Firstly, new spawning grounds have recently been discovered for other bluefin tunas. Atlantic bluefin tuna spawning was thought to be restricted to the Gulf of Mexico and Mediterranean Sea, but larvae were recently collected in the northern Caribbean, Mid-Atlantic Bight, and Bay of Biscay [2, 13, 14]. In the North Pacific, PBF larvae were recently reported for the first time in the Kuroshio Current off eastern Japan [4]. Secondly, the CCS was anomalously warm during a marine heatwave in 2014–2016 [15] and SSTs stayed above average through 2019. This may have increased the thermal habitat suitable for PBF spawning and larval development. Lastly, fishers and spotter plane pilots reported behavior they considered consistent with spawning (e.g., large schools aggregating at the surface and unwilling to take bait) off southern California and northern Baja California. Previous studies have shown the high potential for at-sea observations from industry professionals and interested citizens to inform science and conservation (e.g. [16, 17]). PBF are intensively managed and currently overfished [8]. The discovery of additional spawning areas would have important implications for stock assessment, conservation, and management (e.g., [13]).

In this study, we combined historical data, fishery-dependent observations, and biological sampling to evaluate the potential for PBF spawning in the CCS. Our primary objective was to examine the gonads of large PBF for evidence of maturity and active spawning. In addition, we used multi-decadal ichthyoplankton collections to determine whether larval PBF may have been collected in the CCS previously. Lastly, we used satellite SST and fishery-dependent datasets to define overlap between PBF caught in purse seines and potentially suitable spawning habitat.

## Materials and methods

We used biological knowledge of PBF and the two other bluefin tuna species (*T*. *thynnus*, *T*. *orientalis*) to define our study area. While most bluefin tuna larvae have been collected where SSTs exceed 24°C, [6] reported female PBF in spawning condition in the Sea of Japan where temperatures were as cool as 19.3°C, and [18] collected Atlantic bluefin tuna larvae in the Mediterranean Sea down to 20.5°C. We thus considered areas with SSTs > 20°C in the eastern North Pacific to be potential spawning habitat. Bluefin tuna spawning grounds are also distinct from those of other tunas in that they are concentrated in semi-enclosed seas and offshore continental shelf environments, rather than the open ocean [2]. Decades of ichthyoplankton surveys (e.g. [19–21]) have not recorded any bluefin tuna larvae more than 1,000 km from a major landmass, and so we restricted our study area to waters within approximately 1,000 km of the North American coast. Lastly, more than 60 years of purse seine and pelagic longline fisheries data collected by the Inter-American Tropical Tuna Commission (see below) have not recorded PBF of any size south of approximately 20°N within 1,000 km of land. The spatiotemporal coverage of sampling for gonads and for ichthyoplankton in relation to these constraints is shown in S1 Fig., and each aspect of sampling within the study area is described in more detail below.

### Gonad sampling

**Fig 1:**
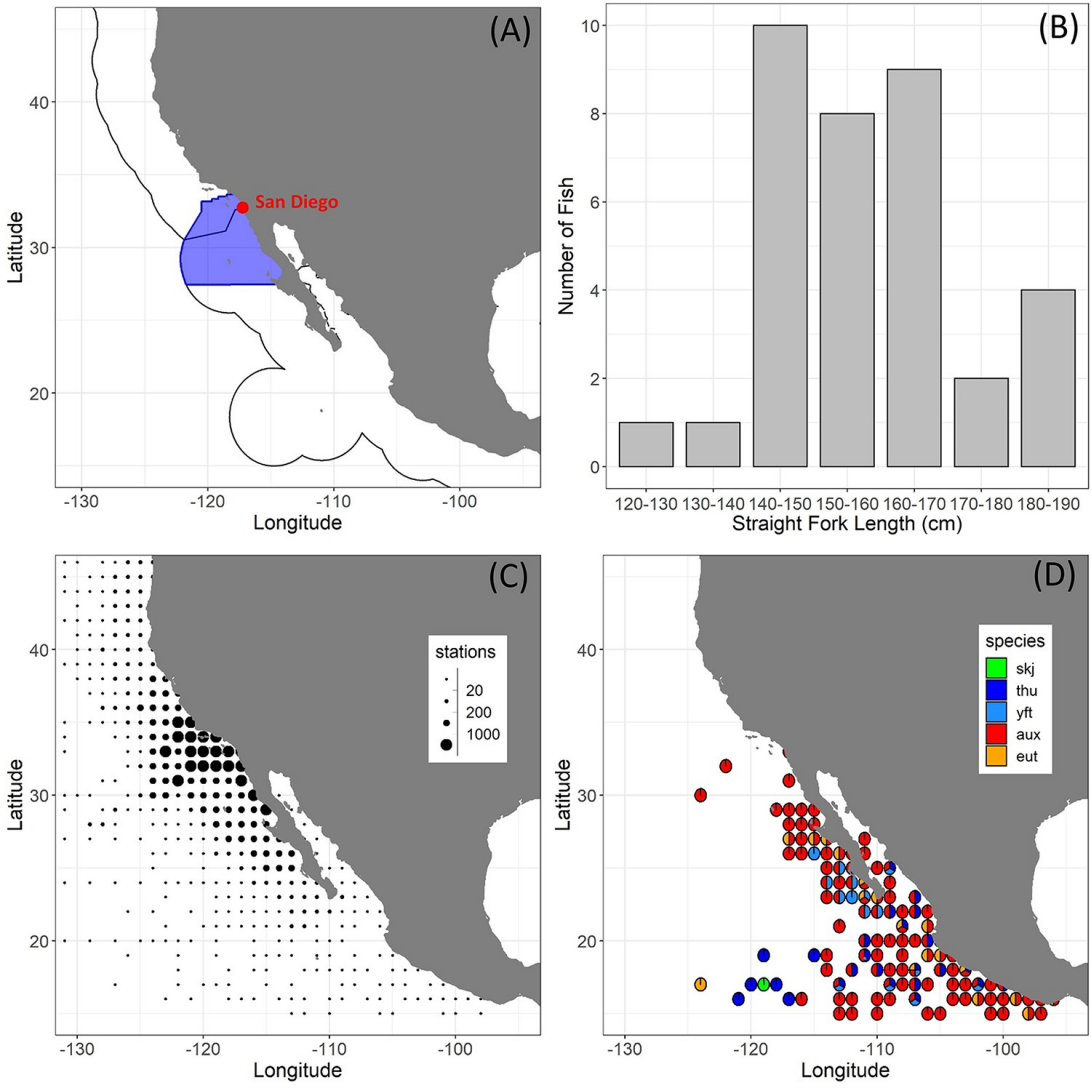
(A) Collection area for Pacific bluefin tuna obtained from the Commercial Passenger Fishing Vessel fleet in 2015–2019 (blue), with U.S. and Mexico Exclusive Economic Zone boundaries also shown, (B) Length distribution of sampled fish, (C) ichthyoplankton sampling stations aggregated to 1 degree resolution from the CalCOFI and IMECOCAL sampling programs (1951–2020), (D) locations where presence of tuna larvae was recorded in either CalCOFI or IMECOCAL samples across all sampled years. “skj” = *Katsuwonus pelamis*, “thu” = *Thunnus* spp. larvae not identified further, “yft” = *Thunnus albacares*, “aux” = *Auxis* spp. larvae, “eut” = *Euthynnus lineatus* larvae.

We collaborated with local fishers and fish processors to collect heads and gonads from 36 female PBF caught during the recreational fishing season (May—November) from 2015 to 2019. Precise fish catch locations were not available, but we defined the spatial extent of the fleet using logbook data from the California Department of Fish and Wildlife (Fig 1). Fish fork length (FL) was estimated from operculum length using a published regression for the southern CCS [22], and estimated ages were generated based on the length-at-age equation provided in [7]. An approximately 1 cm section from the central portion of one of the gonads was sampled from each fish and preserved in 10% neutral buffered formalin. A small section of preserved ovarian tissue from each gonad sample was embedded in paraffin, sectioned at approximately 4μm, and stained with hematoxylin followed by eosin counterstain (Reveal Biosciences, San Diego, CA, USA).

The histological classification of the ovaries was based on [3, 23] and simplified to focus on distinguishing between spawning (e.g., spawning capable, actively spawning) and non-spawning females. Each ovary was assessed for the most advanced stage of oocytes: (1) unyolked (i.e., previtellogenic), (2) early yolked, (3) advanced yolked, (4) migratory-nucleus stage, or (5) hydrated. Ovaries were also examined for signs of atresia. Assignment of imminent, active, or prior spawning requires the presence of advanced yolked oocytes or atresia in yolked oocytes [3]. Females with ovaries containing only unyolked or early-yolked oocytes and with no signs of atresia were classified as inactive.

### Ichthyoplankton

Ichthyoplankton data were sourced from the CalCOFI (1951–2020 via https://coastwatch.pfeg.noaa.gov/erddap/tabledap) and IMECOCAL (1997–2017) sampling programs [24, 25]: Fig 1. Occurrences of scombrid larvae from the Thunnini tribe (genera *Auxis*, *Euthynnus*, *Katsuwonus*, *Thunnus*) were considered to represent potentially suitable tuna spawning habitat. SSTs at sampling stations with and without tuna larvae were compared. Where no *in situ* surface or near-surface (shallower than 5m) temperature was available, we extracted SST from the 0.25 degree NOAA high-resolution blended daily analysis [26]. This resulted in 43,753 separate ichthyoplankton sampling stations with SST data available.

### Fishery-dependent data

The distribution of PBF in the southern CCS was approximated using 1 degree aggregated purse-seine catch data collected by the Inter-American Tropical Tuna Commission. We used data from sets not associated with dolphins or floating objects, reported between 1982 and 2020. SST data were extracted at each 1x1 degree location for each year and month using the NOAA Optimum Interpolation monthly analysis. The presence of PBF in purse seine sets was then quantified with respect to both geography and SST.

## Results

**Fig 2:**
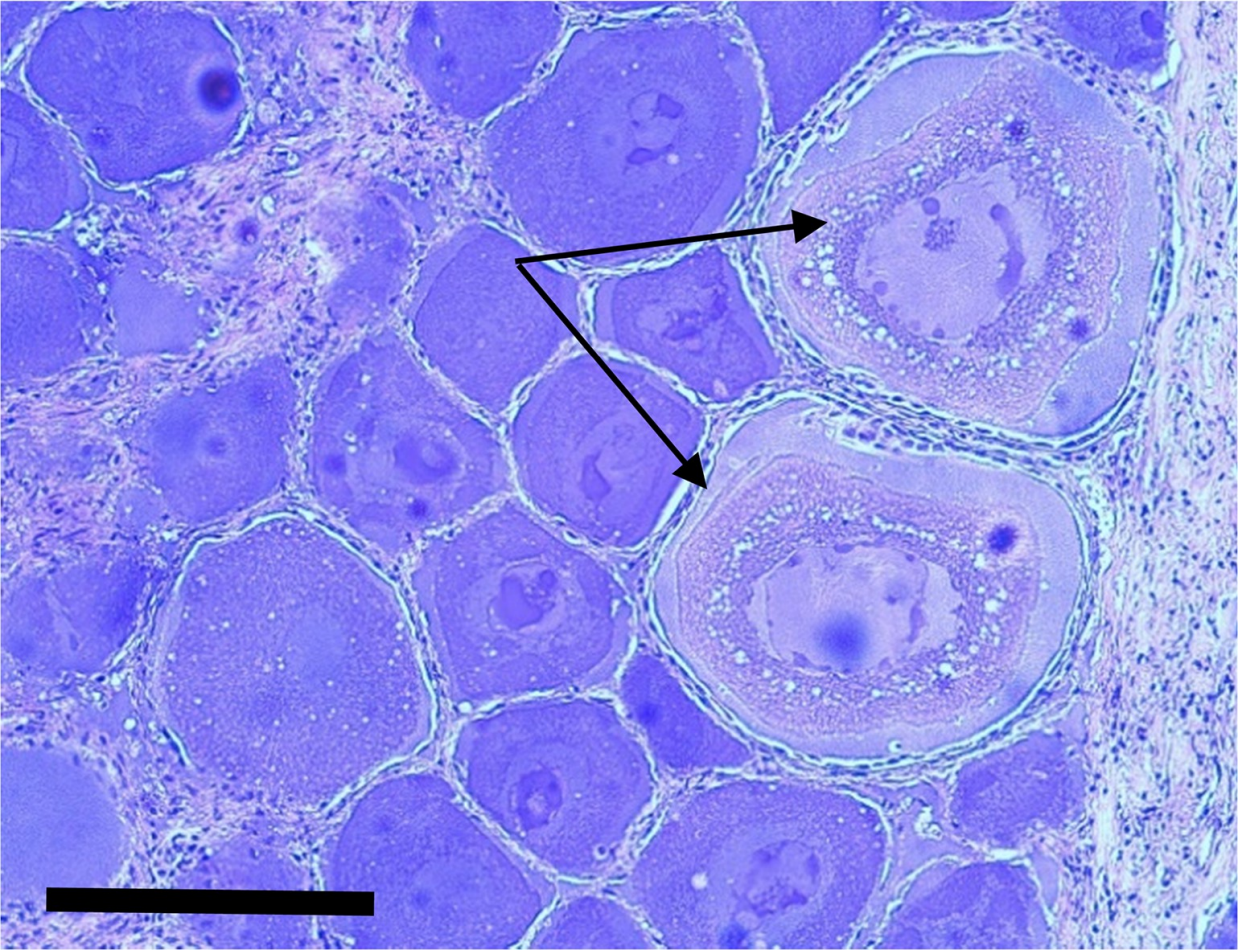
Ovarian tissue section from a 157 cm FL PBF, estimated age 5 (40X magnification). Note the two cortical alveolar oocytes indicated by the arrows. The black scale bar represents 100 μm.

Sampled females ranged in length from 125 to 188 cm FL, and from 3–8 years old (Table 1, S1 Table). Histological analyses of the ovarian tissues revealed no evidence of imminent, active, or prior spawning. The ovaries of all 36 females contained only previtellogenic oocytes (e.g., oogonia, perinucleolar, cortical alveoli). Ovaries from two females (148 and 157 cm FL, both 5 years old) collected in July of 2015 contained a few cortical alveolar oocytes (Fig 2, S1 Table). No atresia was observed.

**Table 1:** Sample sizes, fork length (cm) and estimated age ranges (years), and number of female PBF classified by developmental stage. Note that all data are included in S1 Table.

Size Range cm (mean)	125–188 (158)
Estimated Age Range	3–8
Unyolked	34
Cortical Alveolar	2
Early-Yolked	0
Atresia	0
**Total**	**36**

Ichthyoplankton sampling was most concentrated off southern California and Baja California but included stations throughout the CCS (Fig 1). Among the 43,753 stations sampled since 1951, 261 contained tuna larvae. *Auxis* spp. were the most abundant, and distributed the furthest north, with 2936 larvae collected from 233 stations. Of 86 *Thunnus* spp. larvae recorded, 26 were Yellowfin Tuna (*T*. *albacares*), and 60 were not identified past genus. Larval taxonomist notes from the times of collection show that these 60 larvae were either too small or in too poor a condition to identify to species. However, none resembled PBF larvae (which can usually be separated visually from other *Thunnus* spp. larvae using pigment patterns: [27]). All *Thunnus* larvae were collected south of 26°N (Fig 1). Twenty-one Black Skipjack (*Euthynnus lineatus*) larvae and 1 Skipjack tuna (*Katsuwonus pelamis*) larvae were collected south of 27°N.

**Fig 3:**
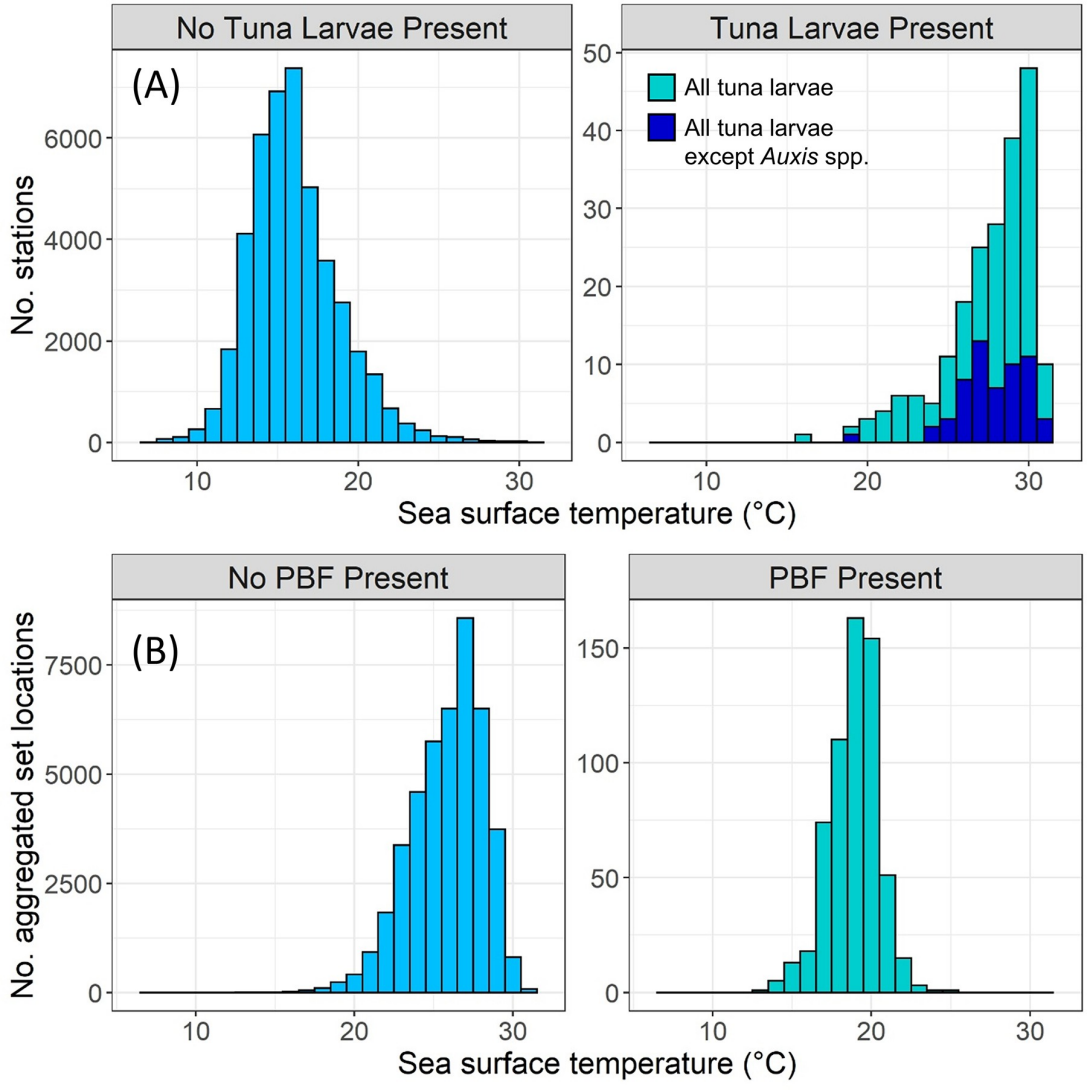
(A) Histograms of sea surface temperature at all CalCOFI and IMECOCAL stations without (left) and with (right) tuna larvae recorded. In situ SST was used if available, otherwise the NOAA high-resolution blended daily analysis was used instead. (B) Histograms of sea surface temperature at all 1x1 degree monthly purse seine fishing locations without (left) and with (right) PBF recorded. SST was extracted from the NOAA Optimum Interpolation Sea Surface Temperature monthly analysis.

**Fig 4:**
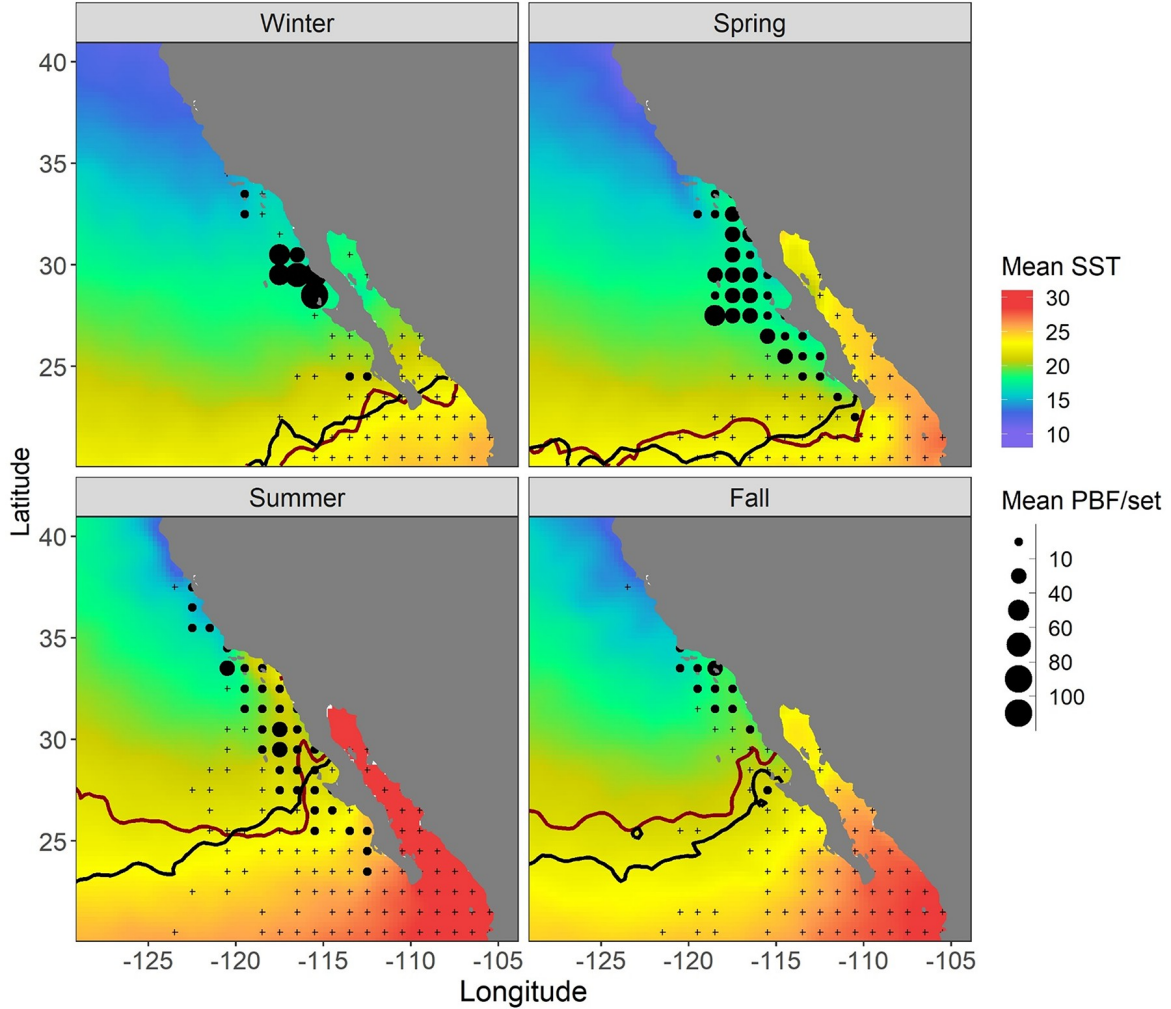
Mean monthly SST by season (winter is January through March, spring is April through June etc.) for 2015 through 2019. The dark red line shows the position of the maximum 24°C isotherm for all months and years 2015–2019. The black line shows the position of the maximum 24°C isotherm for all months and years 1982–2012, for comparison. Mean PBF/set at aggregated 1x1 degree resolution for years 1982–2020 is also shown, to approximate the geographical distribution of PBF in the southern CCS. Black crosses indicate zero PBF catch.

Only 4 tuna larvae were recorded where SSTs were <20°C (Fig 3). Of 69 larvae collected at 20–24°C, 67 were *Auxis* spp. *Thunnus* spp. larvae were restricted to SSTs > 23.7°C. Conversely, PBF were mostly caught by purse seines when monthly SSTs at fishing locations were < 23°C (Fig 3). During 2015–2019, waters >24°C were located to the south of Baja California during winter and spring, with some warmer waters reaching the southern peninsula in summer and fall (Fig 4). Calculation of the maximum SST recorded in each pixel during 2015–2019 showed that some southern Baja waters were > 24°C during summer and fall. Despite recent heatwave conditions, the position of the 24°C isotherm was not markedly different in the southern CCS in 2015–2019 than in prior years (1982–2012) (Fig 4). Catches of PBF in purse seines were low off southern Baja California during these warmer seasons, consistent with their association with cooler SSTs (Fig 3). The overlap between PBF and *Thunnus* spp. larvae was thus minimal in both geographic and thermal space.

## Discussion

This study combined new biological collections with historical data to examine the potential for PBF spawning in the southern CCS. Based on histological evaluations of ovarian tissues, there was no evidence that female PBF sampled were actively spawning, or had previously spawned, in this region of the eastern Pacific. Two females had very few cortical alveolar oocytes present, suggesting they may have been in a very early “developing” phase towards sexual maturity [28]. However, it is unknown whether these animals may actually mature and spawn in the CCS or will resorb those oocytes and forego maturation until they migrate to the western Pacific. While we found no definitive evidence of recent or imminent spawning among sampled females, atresia and resorption of yolked oocytes and post-ovulatory follicles in tunas can occur over a period of a few days [29, 30]. Therefore, we cannot completely eliminate the possibility that some of the sampled females may have previously spawned.

Gonad sampling was spatially restricted due to our reliance on U.S. fisheries for samples. However, ichthyoplankton and fishery-dependent data covered both U.S. and Mexican waters across multiple decades. These data showed minimal geographic and thermal overlap between larval tuna habitat and PBF fishing habitat. Although purse seine data may be biased by seasonal fishing closures, they are consistent with previous tagging studies. Tagged PBF rarely moved south of Baja California, and did not occupy waters > 24°C in the CCS [9, 31]. Based on lengths reported at release and recapture, these tagged PBF were likely a mix of juvenile and adult fish (based on [6]). A more recent study during the 2014–2016 marine heatwave [32] recorded PBF where SSTs were as high as 24.5°C, but the majority of occurrences were still at < 23°C.

In contrast, *Thunnus* larvae were primarily associated with SSTs > 23–24°C, as has been reported for other regions [2]. In the CCS, these temperatures are largely restricted to southern Baja California during summer and fall. The 60 *Thunnus* spp. larvae not identified to species in ichthyoplankton collections are thus very unlikely to be PBF. In addition, conditions in the CCS are not typical of those on bluefin tuna spawning grounds. Spawning areas in the western North Pacific, Atlantic, and Indian Oceans are oligotrophic with warm SSTs [2, 5]. In addition, most spawning grounds are linked to juvenile foraging habitats via poleward boundary currents, which transport small juveniles to cooler, more productive waters at higher latitudes. For example, the Kuroshio Current transports juvenile PBF from the Nansei Islands towards coastal Japan, the Gulf Stream takes juveniles north to the Mid-Atlantic Bight [2], and the Leeuwin Current transports juveniles to southern Australia [1]. In contrast, the CCS is a productive upwelling boundary current, which flows equatorwards towards the subtropics.

If larger PBF in the CCS are not functionally mature and not spawning, then the reasons for their remaining in the eastern North Pacific are unclear. They may be exploiting optimal foraging habitat, and delaying their return to natal spawning grounds in the western Pacific. We note that plasticity in age at maturity of bluefin tuna is also seen in the North Atlantic. Some juvenile Atlantic Bluefin Tuna migrate from the Mediterranean Sea to western Atlantic foraging grounds, remaining there until they are up to 9 years old before returning to the Mediterranean to spawn [33]. In contrast, fish that remain in the Mediterranean can start spawning as early as 3 years old [34].

Although a “negative” result, we consider our findings important to report. Several new spawning grounds for Atlantic bluefin tuna were described only after exploration of poorly sampled regions [13, 14]. These studies suggest that spawning was not new to these areas but rather had gone unnoticed due to a lack of sampling. In addition, where limited historical samples did show Atlantic bluefin tuna larvae outside known spawning grounds [35], these were largely dismissed as advection events: a conclusion which now seems highly unlikely based on more recent analysis of drifter tracks [13]. The importance of re-visiting historical data and re-examining accepted paradigms on spawning distributions in species such as bluefin tunas should thus be apparent.

As climate change leads to further ocean warming, it will be essential to distinguish between environmentally-driven shifts in spawning areas, and discovery of existing spawning grounds through additional sampling. Although our sampling coverage was imperfect, and could be improved with additional samples from southern Baja California, a combination of multiple data streams suggest that PBF are not likely to be spawning in the CCS. The drivers and ecological consequences of the presence of larger, older PBF in the CCS in recent years thus require further investigation.

## Supporting information

S1 Table Pacific bluefin tuna metadata and results of the histological analyses of female gonads. Atresia of yolked oocytes was not present in any female.
(PDF)

S1 Fig Sampling effort within the study area. Color scale shows mean monthly sea surface temperature for years 2015–2019 from the NOAA Optimum Interpolation monthly analysis. The blue contour represents 20°C (hypothesized lower limit for spawning activity), while the dark red represents 24°C (coolest temperature at which bluefin tuna larvae are usually collected). Black dots show ichthyoplankton sampling effort as total number of stations from the CalCOFI and IMECOCAL programs, 1951–2020. The blue polygon shows the area within which PBF gonads were obtained (sampling covered the months of May through November, 2015–2019). Areas more than 1,000 km from the nearest major landmass are masked in white. The subpanel labels denote month of the year.
(TIF)
