# Supplementary material for: Recent and historical data show no evidence of Pacific bluefin tuna reproduction in the southern California Current system
Source: PLoS One. 2022 May 26;17(5):e0269069. doi: 10.1371/journal.pone.0269069 (PMC9135278; doi:10.1371/journal.pone.0269069)
Supplement: S1 Table — Atresia of yolked oocytes was not present in any female. (PDF) [file pone.0269069.s001.pdf]

Supplementary Table 1: Pacific bluefin tuna metadata and results of the histological analyses of female gonads. Atresia of yolked oocytes was not present in any female.

Sample #	Sex	FL	Date Sampled	Most advanced stage of oocytes
3130	F	126	6/25/2019	unyolked
3146	F	150	6/25/2019	unyolked
3159	F	149	7/28/2019	unyolked
754-06	F	182	5/4/2019	unyolked
797-11	F	157	6/15/2015	cortical alveolar oocytes
797-67	F	168	7/16/2015	unyolked
797-85	F	157	7/28/2015	unyolked
797-88	F	160	7/28/2015	unyolked
797-89	F	148	7/28/2015	cortical alveolar oocytes
797-90	F	149	7/28/2015	unyolked
797-92	F	158	7/28/2015	unyolked
846-25	F	147	8/18/2017	unyolked
847-69	F	144	9/1/2017	unyolked
850-20	F	144	9/20/2017	unyolked
850-37	F	189	9/20/2017	unyolked
851-17	F	140	7/16/2017	unyolked
851-60	F	182	7/29/2017	unyolked
859-69	F	136	6/28/2017	unyolked
859-70	F	165	6/28/2017	unyolked
859-86	F	163	6/18/2017	unyolked
880-93	F	149	11/20/2017	unyolked
880-94	F	153	11/20/2017	unyolked
904-66	F	159	6/19/2018	unyolked
904-69	F	143	6/21/2018	unyolked
904-71	F	155	6/21/2018	unyolked
904-72	F	168	6/21/2018	unyolked
904-75	F	161	6/21/2018	unyolked
904-76	F	165	6/21/2018	unyolked
913-54	F	165	7/17/2018	unyolked
914-03	F	173	7/23/2018	unyolked
914-09	F	166	7/23/2018	unyolked
914-60	F	167	8/1/2018	unyolked
914-83	F	183	8/6/2018	unyolked
925-42	F	171	9/14/2018	unyolked
929-100	F	157	10/28/2018	unyolked
913-59	F	169	7/17/2018	unyolked
