# Supplementary figures and images for: Recent and historical data show no evidence of Pacific bluefin tuna reproduction in the southern California Current system
Source: PLoS One. 2022 May 26;17(5):e0269069. doi: 10.1371/journal.pone.0269069 (PMC9135278; doi:10.1371/journal.pone.0269069)

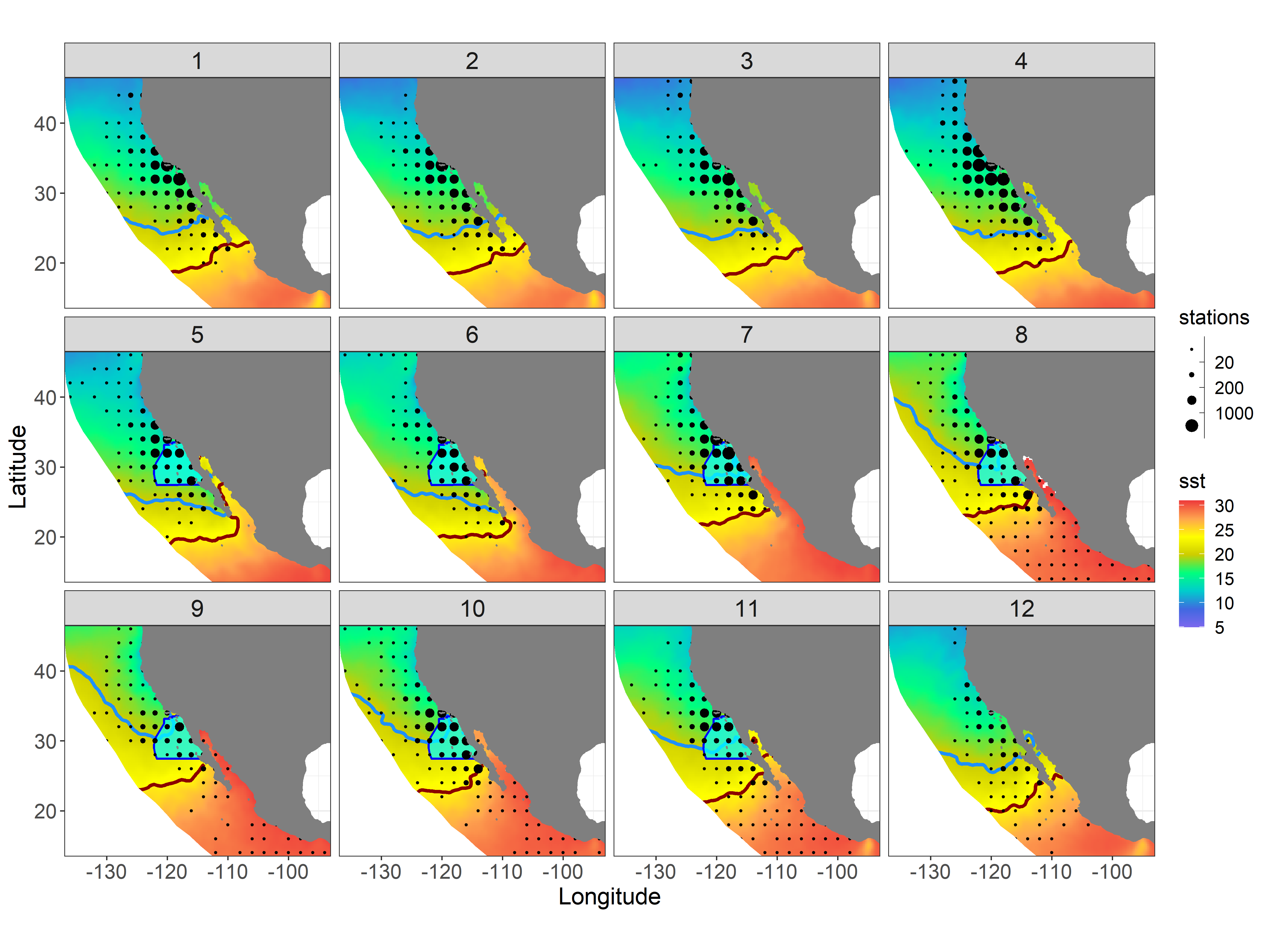

Supplement: S1 Fig — Color scale shows mean monthly sea surface temperature for years 2015–2019 from the NOAA Optimum Interpolation monthly analysis. The blue contour represents 20°C (hypothesized lower limit for spawning activity), while the dark red represents 24°C (coolest temperature at which bluefin tuna larvae are usually collected). Black dots show ichthyoplankton sampling effort as total number of stations from the CalCOFI and IMECOCAL programs, 1951–2020. The blue polygon shows the area within which PBF gonads were obtained (sampling covered the months of May through November, 2015–2019). Areas more than 1,000 km from the nearest major landmass are masked in white. The subpanel labels denote month of the year. (TIF) [file pone.0269069.s002.tif]
